# A rare case of monostotic Melorheostosis of right ulna presenting with chronic forearm pain - Case report

**DOI:** 10.1016/j.ijscr.2024.110603

**Published:** 2024-11-13

**Authors:** Thinley Ugyen, Letho Letho

**Affiliations:** Department of Orthopedics, Jigme Dorji Wangchuck National Referral Hospital, Thimphu, Bhutan

**Keywords:** Melorheostosis, Right ulna, Chronic pain, Case report

## Abstract

**Introduction:**

Melorheostosis is extremely rare non-cancerous lesion of bone mainly affecting the long bones and soft tissues. The incidence is 0.9 per million population. It affects male and female equally and reported among children and early adulthood. Common symptoms include pain, deformity and restricted range of movement of affected joint.

**Case presentation:**

A 28-year-old female presented with pain at right forearm for nine months. The pain was of insidious onset and progressive in nature with severity of VAS 7/10. On examination, no gross deformity of right forearm was noted, no tenderness along the bones and range of movement of right elbow and wrist joints were within normal limit. Radiograph of right forearm revealed sclerosis of entire right ulna with dripping candle wax appearance with bowing deformity of ulna. She underwent osteoplasty of right ulna and biopsy. Oral bisphosphonate was started.

**Discussion:**

Due to the rarity of the condition, there is still no standard guideline of the treatment of Melorheostosis, the widely practiced mode of treatment is symptomatic with analgesics, contracture release and deformity correction in extreme cases. If the diagnosis is doubtful it is important to rule out other sinister causes such as infection and malignancy. Biopsy is indicated only to confirm diagnosis.

**Conclusion:**

Early diagnosis of Melorheostosis still remains challenge due to paucity of the condition. Melorheostosis should be included in one of the differential diagnosis of any hypersclerotic conditions of bone.

## Highlights

• Melorheostosis is extremely rare condition characterized by dysplasia and hypersclerosis of bone.
• Diagnosis is based on typical ‘flowing candle wax’ appearance in radiographs
• Usually presents with pain, restricted movement of joints and deformity
• Treatment is usually directed towards symptom relief either through medical or surgical interventions.

## 1 Introduction

Melorheostosis derives its meaning from Greek word ‘melos’: limb, ‘rhein’: to flow, ‘ostos’: bone. It is nonhereditary bone pathology characterized by endochondral and intramembranous ossification of bone [1]. It was first described by Leri and colleague in 1922 and it was named as Leri's disease. It predominantly affects the unilateral appendicular skeleton mainly the long bones of lower limbs [2]. It is a rare benign disease with worldwide prevalence of 1/1000, 000 and affects males and females equally [3].

The exact cause of Melorheostosis still remains mystery. Genetic mutation of MAP2K1 and SMAD3 genes were implicated for dripping candle wax form and endosteal form respectively [4]. Melorheostosis is associated with various conditions like nephrotic syndrome, linear scleroderma, hyperpigmentation, fibroma, lipomatosis and retroperitoneal fibrosis [5].

Clinical presentation will depend on site, extent of bony and soft tissue involvement. Majority of the condition is detected incidentally during imaging for other issues. Pain is the most common clinical symptom [6]. If there is breach of joints, it can cause stiffness of affected joint. If there is compression of nerve it can cause pain, numbness, tingling sensation and weakness of affected myotome [7].

The diagnosis of Melorheostosis is established mostly through imaging modalities like plain radiography, CT, MRI and bone scans. Histopathological diagnosis through biopsy is performed only in case of suspicious or sinister lesions and as a part of surgical intervention. The typical radiographic appearance of Melorheostosis is irregular hyperosteosis that predominantly affects outer cortex, in certain cases it involves cancellous bone and can appear as completely radiopaque or mixed pattern.

There is no medical treatment to modify or cure the disease. Physical therapy still remains crucial treatment approach to relief pain. Surgical interventions are warranted only when there are functional deformities, contracture of joints and in case of neurovascular compressions.

## 2 Method

The information in this case report is obtained from retrospective chart review and is reported in line with SCARE guideline [8]. Patient's informed written consent was obtained to use data for publication of this case report.

## 3 Case report

A 28-year-old female presented with chief complaint of pain at right forearm for nine months duration. The pain was of insidious onset, progressive dull pain with VAS of 7/10. Pain was constant and not aggravated by forearm, elbow and wrist movements. Pain improved with analgesia and there was no night pain. She denied any history of trauma, fever or other constitutional symptoms. She also denied family history of malignancy and medical comorbidities.

Physical examination revealed prominence of medial aspect of shaft of right forearm. There was no tenderness along the ulna and radius. Tinel sign was negative. Forearm, wrist and elbow range of movements were within normal limit. Distal neurovascular status was intact.

**Fig. 1:**
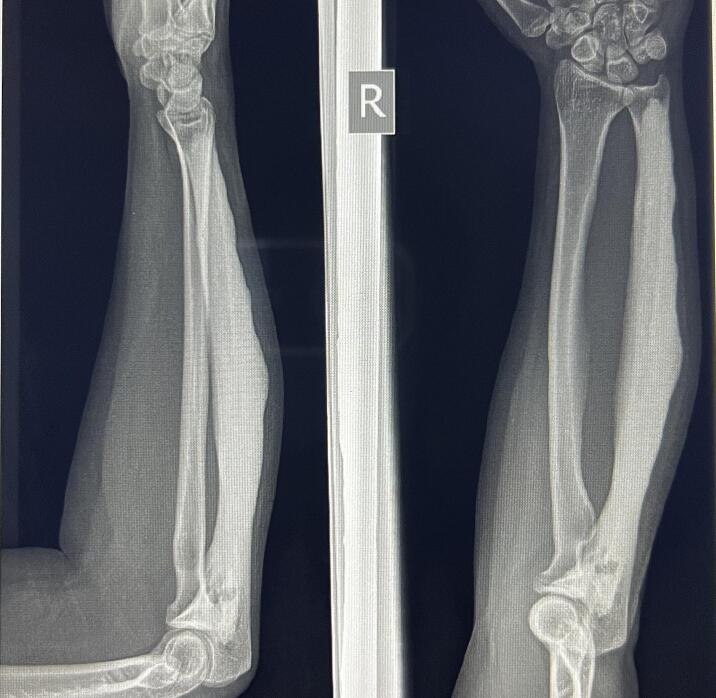
Radiograph of right forearm anteroposterior and lateral views showing cortical thickening and hyperosteotic lesion of right ulna with ‘dripping candle wax’ appearance and radial bowing of ulna.

Radiograph of right forearm AP (anteroposterior) view revealed homogenous hyperosteosis of entire ulna from olecranon till the distal end (Fig. 1). The ossification is maximum at the mid shaft of ulna. Dripping candle wax appearance was noted at distal third of ulna. There was no radiographic evidence of ossification of surrounding soft tissues and the adjacent joints.

In order to rule out common etiologies, a blood inflammatory markers test was performed. The laboratory investigation revealed WBC-4.75 (4.21–10.29 × 10×3/μL), ESR- 60 (0.0–15 mm/h) and CRP- 1.6 (0.00–6 mg/L).

She was treated with physical therapy and a course of ibuprofen for initial one month. Her pain improved during the course of analgesics but symptom recurred once it is stopped.

Since her chief complaint was progressive pain of right forearm and radiograph showed maximum ossification at mid shaft of ulna causing local prominence and pain, patient was offered to undergo osteoplasty of right ulna and soft tissue release and biopsy for histopathological confirmation of diagnosis.

**Fig. 2:**
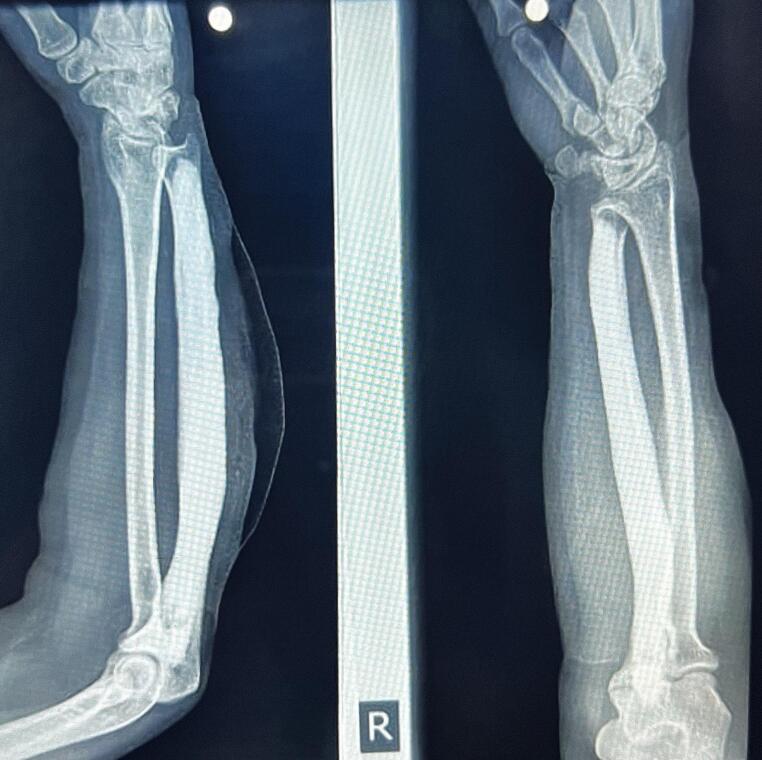
Postoperative radiograph of right forearm reshaped to normal anatomical contour of ulna after osteoplasty.

Patient was placed in supine position with right forearm placed over mayo table. Tourniquet was applied and shaft of right ulna was approached through posterior approach. After the soft tissue dissection, ulna shaft was exposed and periosteum was stripped using periosteal elevator. The osteoplasty of prominent ossified bone was performed using oscillating saw and the adequacy of osteoplasty was checked under fluoroscopy (Fig. 2). The stripped bone samples were sent for histopathology. Intraoperatively there was minimum soft tissue adhesion at the bony prominence. Wound was irrigated and routine closure was done. No splints were applied and patient was allowed to do elbow, wrist and forearm range of movements during immediate postoperative period. She was discharged on postoperative day two with oral bisphophonate to be continued till one month follow up.

At one month postoperative follow up, her pain has improved from VAS 7/10 to 3/10. The range of movement of wrist, elbow and forearm was status quo. She was advised to continue bisphosphonate for next six months. Histopathology report revealed large area of sclerotic bone trabeculae and haversian system of different calibers and occasional areas of bone marrow fibrosis.

## 4 Discussion

We present the first reported case of Melorheostosis from Bhutan. Involvement of upper limb makes it more unique as majority of Melorheostosis are reported in long bones of lower limbs. Due to benign and rare nature of this condition, diagnosis is usually missed during the initial visits. When we reviewed the radiograph during her first visit, the first thing that we wanted to rule out was infection such as chronic osteomyelitis (COM). With no history of fever and absence of radiological features of COM such as involucrum, sequestrum and cloaca and also homogenous involvement of entire ulna, COM was unlikely pathology. Benign bone tumors such as osteoma usually presents with focal lesion rather than entire involvement of affected bone. Though dripping candle wax feature is typical of Melorheostosis, it is important to rule out other common pathologies which can depict similar radiographic appearance. CT scan can help to evaluate the precise extent of the bony lesion especially when there is clinical evidence of soft tissue compression such as blood vessels or nerves. MRI scan is particularly useful for evaluation of surrounding soft tissue involvement like tendons, muscles or nerves etc. [10]. In our case clinically she had no evidence of soft tissue involvement and her chief complaint was local pain. Therefore we did not advise her to undergo CT or MRI imaging as the extent of the bony lesion was clear from the radiograph.

Reassuring patient is very important part of management. Patient should be explained about the benign nature of the condition and lack of proven specific treatment to cure the disease. The drugs which targets process of bone remodeling and proliferation such as bisphosphonates are often recommended. However there is still no proven evidence to validate its efficacy [9].

Clinical symptoms have considerable variation with mild pain to severe deformities of affected limbs. Pain is the most common complaint. In our case patient had progressive pain with mild deformity of forearm with preserved adjacent joints range of movements.

The distribution of lesion is monostotic if one bone is involved or polyostotic if multiple bones are involved. Our case had involvement of only right ulna bone which is very rare.

Commonly performed surgical interventions such as osteotomy, tendon lengthening, contracture release, excision of soft tissue mass and deformity corrections are reserved only when there is involvement of joint, soft tissues and presence of functional deformities.

In our case, we believe that the source of pain is due to local mass effect of progressively expanding bony lesion. In the literature it was reported that the pain in Melorheostosis is predominantly due to increase in vascularity, activation of pain receptors, increase osteoclastic bone resorption and local compression of soft tissue due to excessive bone growth [10]. Since non operative treatment failed to relieve her symptom, she underwent osteoplasty to reduce the local mass effect of abnormal bony growth. We believe that by stripping periosteum during osteoplasty, the pain from hypervascularity can be minimized in addition to reducing the pain due to local mass effect. Post operatively her symptom improved and at two months follow up she was pain free without medications.

## 5 Conclusion

We present a very rare case of Melorheostosis of right ulna with pain as chief complaint. Osteoplasty of bony prominence can help relieve pain by reducing the local mass effect to surrounding soft tissues.

## Abbreviations

CBC: Complete Blood Count
CRP: C-reactive Protein
ESR: Erythrocyte Sedimentation Rate
MRI: Magnetic Resonance Imaging
VAS: Visual analogue score

## Author contribution

Ugyen Thinley: Study concept, manuscript writing, data collection

Letho: Manuscript writing, literature review, data collection

## Consent

Written informed consent was obtained from the patient for publication and any accompanying images. A copy of the written consent is available for review by the Editor-in-Chief of this journal on request.

## Ethical approval

The ethical approval to publish case report is not required by the Institutional Review Board of Khesar Gyalpo University of Medical Sciences of Bhutan.

## Guarantor

Tshering Wangchuk.

## Research registration number

None.

## Funding

No funding was sought for this study.

No source of funding was sought for conducting this study.

## Conflict of interest statement

Authors declare no conflict of interest related to this case report.
